# A Reference Hand-Book of the Medical Sciences

**Published:** 1901-05

**Authors:** 


A REFERENCE HAND-BOOK OF THE MEDICAL SCIENCES.—Embracing the entire range of scientific and practical medicine and allied science. By various writers. A new edition, completely revised and rewritten. Edited by Albert H. Buck, M. D., of New York City. Volume I. Illustrated by numerous chromo-lithographs and four hundred and ninety-eight fine half-tone and wood engravings. 799 pages. Price per volume, $6.50. New York: William Wood & Co. 1900.

The first edition of the Reference Hand-Book was finished in 1887, three years having been spent in its preparation. Seven years later a supplementary volume was issued, which was to take the place of a revision. Medical progress has been so great since then that a thorough rewriting and revision were considered necessary, hence the new edition. It is not stated how many volumes will compose the present work, nor when it will be completed. The profession will hope that less than three years will be required for its completion.

This is a book that will be looked forward to with greater interest than is usual. Covering the entire range of scientific and practical medicine, as well as the allied sciences, makes the work indispensable and necessary to every practitioner's library. The subject matter is arranged in alphabetical order, and the articles are prepared by experts with great care. If one take the time to compare the present volume with the first volume of the old edition, he will find that nearly half of the old matter has been supplied by new, and that the cuts also show the signs of advances in medicine. Taking the work spent upon this volume as a criterion by which to judge the forthcoming seven or eight volumes, which will complete the set, the Reference Hand-Book will be the most popular work published at the beginning of the new century.

